# Increased mortality in chronic hypoparathyroidism: a nationwide cohort study in Sweden

**DOI:** 10.1530/EC-25-0450

**Published:** 2026-07-07

**Authors:** Wafa Kamal, Ylva Trolle Lagerros, Michael Mannstadt, Tim Spelman, Olle Kämpe, Sigridur Björnsdottir

**Affiliations:** ^1^ Department of Molecular Medicine and Surgery, Karolinska Institutet, Stockholm, Sweden; ^2^ Department of Endocrinology, Metabolism and Diabetes, Karolinska University Hospital, Stockholm, Sweden; ^3^ Department of Medicine, Huddinge, Karolinska Institutet, Stockholm, Sweden; ^4^ Endocrine Unit, Massachusetts General Hospital and Harvard Medical School, Boston, USA; ^5^ Department of Clinical Neuroscience, Karolinska Institutet, Stockholm, Sweden; ^6^ Department of Medicine, Solna, Karolinska Institutet, Stockholm, Sweden

**Keywords:** hypoparathyroidism, prevalence, mortality, epidemiology, Sweden

## Abstract

**Context:**

Previous studies on mortality outcomes in patients with chronic hypoparathyroidism have reported inconsistent results.

**Objective:**

To assess all-cause and cause-specific mortality and estimate the prevalence of chronic hypoparathyroidism in Sweden.

**Methods:**

Chronic hypoparathyroidism was defined as the combination of ICD-10 diagnoses and long-term dispensations of active vitamin D. Nationwide diagnostic codes, drug dispensation, and mortality data were obtained from the National Patient Register, Prescribed Drugs Register, Cause of Death Register, and the Total Population Register. Mortality was compared between groups using Cox proportional hazards models, adjusting for age and baseline comorbidities.

**Results:**

We identified 1,982 individuals with chronic hypoparathyroidism between 1997 and 2018, yielding a minimum prevalence of 19.4 per 100,000 inhabitants. We randomly selected 19,820 controls matched by age, sex, and county of residence.

After excluding individuals with baseline thyroid cancer and their controls, 1,825 patients and 17,922 controls remained. Patients had higher all-cause mortality than controls (HR: 1.55; 95% CI: 1.40–1.72). Mortality was higher in patients with nonsurgical (HR: 2.16, 95% CI: 1.76–2.65) than postsurgical hypoparathyroidism (HR: 1.39, 95% CI: 1.23–1.58). The strongest associations were observed for endocrine, nutritional, and metabolic disorders (HR: 6.46, 95% CI: 2.43–17.21), followed by infectious diseases (HR: 3.53, 95% CI: 2.07–6.04), genitourinary diseases (HR: 2.40, 95% CI: 1.33–4.33), respiratory diseases (HR: 1.67, 95% CI: 1.23–2.28), and circulatory diseases (HR: 1.47, 95% CI: 1.23–1.77).

**Conclusion:**

Patients with chronic hypoparathyroidism have an elevated risk of all-cause mortality, particularly those with nonsurgical disease. These findings highlight the clinical burden of the disease.

## Introduction

Hypoparathyroidism is a complex endocrine disease characterized by insufficient production or secretion of parathyroid hormone (PTH), leading to several disturbances in mineral metabolism, including hypocalcemia and hyperphosphatemia (1, 2). The prevalence of hypoparathyroidism is estimated to range from 6.4 to 37 per 100,000 individuals in different studies, classifying it as a rare disease (2, 3). The most common cause of hypoparathyroidism is anterior neck surgery, such as thyroidectomy or parathyroidectomy, accounting for approximately 75% of cases (4). Less common etiologies are autoimmune destruction of the parathyroid glands, genetic disorders, and rare infiltrative diseases (4).

Recent advances in the understanding of the clinical consequences of hypoparathyroidism have highlighted significant complications and a decrease in the quality of life of affected patients (5, 6). A recent systematic review identified the most common complications in patients with chronic hypoparathyroidism to be kidney stones, renal insufficiency, arrhythmias, ischemic heart disease, and infections (7). A Swedish study on postsurgical hypoparathyroidism reported increased mortality, while a Scottish study investigating all forms of chronic hypoparathyroidism demonstrated increased mortality rates only in patients with nonsurgical hypoparathyroidism (8, 9). In contrast, nationwide studies in Denmark found no significant association between mortality and postsurgical hypoparathyroidism (10, 11). Similarly, a study from South Korea found no such association in patients with nonsurgical hypoparathyroidism (12).

By using comprehensive data from high-quality national health registers in Sweden, we identified a large, well-defined cohort of patients with chronic hypoparathyroidism receiving conventional treatment. This allowed us to investigate the prevalence of chronic hypoparathyroidism in Sweden, the mortality rates, and specific causes of death, compared to matched controls.

## Methods

### Study design

This nationwide, retrospective cohort study used data from Swedish national health registers, linking individual-level information through unique personal identification numbers (PINs). The PIN is a unique, 12-digit number assigned to each Swedish resident (13). The use of a unique PIN allows an accurate cross-linking of data between different national registries (13). Ethical approval for this nationwide registry-based study was obtained from the Swedish Ethical Review Authority in Stockholm, Sweden (approval number: 2017/476-31/4).

### Data sources

The Swedish healthcare system is universal, ensuring access to healthcare services for all residents. All healthcare providers are mandated to report to the national health registers, which are managed by the National Board of Health and Welfare in Sweden.

#### The Swedish National Patient Register

The Swedish National Patient Register, established in 1964, with nationwide coverage since 1987, includes information on both inpatient and hospital outpatient data since 2001 (13). It does not include data from primary care settings; however, in Sweden, the vast majority of patients with chronic hypoparathyroidism are followed up in hospital outpatient clinics according to Swedish clinical guidelines. The registry contains information such as the PIN and ICD-10 discharge codes (13).

#### The Swedish Prescribed Drug Register

The Swedish Prescribed Drug Register, established in July 2005, provides comprehensive data on all prescribed medications dispensed to the Swedish population (14). It contains information on the medication, prescription details, and patient characteristics, including age, sex, and PIN (14). However, it does not include data on over-the-counter medications, drugs administered during hospital care, and clinical details, such as diagnoses or treatment indications. Prescriptions in Sweden are valid for 12 months, requiring at least annual renewal for continued treatment (15). Since 2002, to qualify for subsidies under the Swedish government-sponsored healthcare system, patients have been receiving a three-month supply at each dispensation (15). Active vitamin D is not available over the counter, while calcium supplementation is, although it is typically prescribed to enable reimbursement through the public healthcare subsidy system.

#### The Swedish Cause of Death Register

The Swedish Cause of Death Register has recorded the cause of death for all individuals who have died in Sweden, including non-residents, since 1952. Since 2012, it also includes deaths occurring abroad among Swedish residents (16). The register provides information about the immediate cause of death, contributory factors, and preceding conditions contributing to the death. However, it does not capture all diagnoses present in the deceased (16).

#### The Swedish Total Population Register

The Swedish Total Population Register contains data on demographic information and dates of death (17).

### Study population

Patients of all ages with an ICD-10 diagnosis of hypoparathyroidism, postsurgical hypoparathyroidism, DiGeorge syndrome, or autoimmune polyglandular failure were identified in the Swedish National Patient Register between January 1, 1997, and December 31, 2018, corresponding to the period following the nationwide implementation of the ICD-10 classification system in Sweden (Supplementary Table S1 (see section on Supplementary materials given at the end of the article)).

Postsurgical hypoparathyroidism was categorized using the ICD-10 diagnosis code, E89.2, recorded at baseline. Procedure codes were not used, as they were not systematically registered in the Swedish National Patient Register prior to 2007.

Chronic hypoparathyroidism was defined as hypoparathyroidism with ongoing conventional treatment, specified as treatment with active vitamin D analogs, with or without calcium supplementation, for more than 12 months after diagnosis. Patients diagnosed prior to the start of the Prescribed Drug Register in 2005 had to receive at least two dispensations of active vitamin D in the first year of the register. Patients diagnosed in 2005 or later had to receive two dispensations of active vitamin D at least 13 months after the date of diagnosis. Hence, only patients with both a relevant diagnosis and treatment with active vitamin D for more than 12 months were included and defined as chronic hypoparathyroidism cases. Patients with fewer than two dispensations of active vitamin D in the final year of follow-up were excluded. To minimize the risk of including misclassified patients with secondary hyperparathyroidism, we excluded patients with pre-existing kidney disease before baseline from our analysis, as ICD-10 codes for secondary hyperparathyroidism are not consistently or reliably reported in clinical practice.

**Figure 1:**
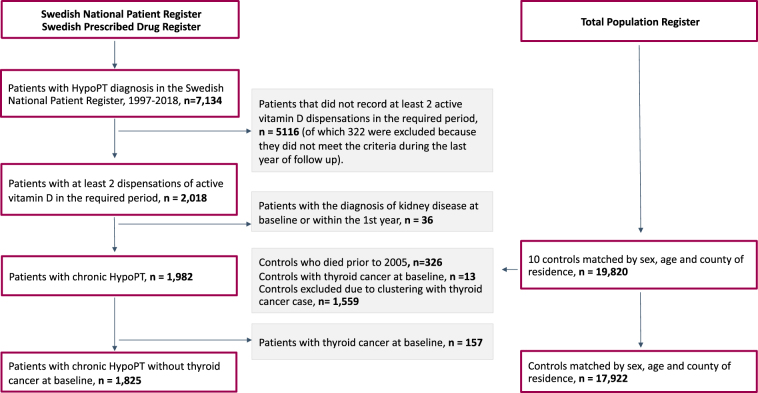
Flow chart illustrating the inclusion and exclusion criteria for patients with chronic hypoparathyroidism and their matched controls in the mortality analyses.

For each case, ten controls were matched by year of birth, sex, and county of residence using the Total Population Register. In the mortality analyses, we excluded 157 patients with baseline thyroid cancer and all their matched controls, as well as an additional 13 controls with baseline thyroid cancer and 326 controls who died before the start of the Swedish Prescribed Drug Register in 2005. The final control cohort, thus, comprised 17,922 individuals (Fig. 1).

Patients and controls with a history of thyroid cancer at baseline were included in the prevalence analysis but excluded from the mortality analyses. This approach was taken because thyroid cancer surgery can lead to postsurgical hypoparathyroidism, which is relevant for estimating prevalence, whereas inclusion of patients with previous thyroid cancer could introduce confounding in the mortality analyses. These exclusions explain the reduction from the initially matched 19,820 controls to the final analytic sample of 17,922 (Fig. 1).

For the cause-specific mortality analyses, the immediate cause of death, as recorded in the Swedish Cause of Death Register, was used in our analysis.

Baseline for all patients with hypoparathyroidism was defined as the date of the first recorded diagnosis of hypoparathyroidism in the National Patient Register (ICD-10 codes provided in Supplementary Table S1).

The end of the follow-up period was defined as either the date of death or the end of the study period, whichever came first.

### Outcomes

The primary outcome was the estimated prevalence of chronic hypoparathyroidism and differences in all-cause mortality between patients with chronic hypoparathyroidism and matched controls.

Secondary outcomes were analyses of cause-specific mortality, differences in mortality by sex, etiology (postsurgical vs nonsurgical), and changes in mortality rate over time. To examine whether mortality rates shifted over time, we performed additional analyses by splitting the follow-up period into three distinct intervals (2005–2009, 2010–2014, and 2015–2018). This approach allowed us to directly compare mortality rates across each interval and identify any trends or changes that may have emerged over the course of the study.

### Statistical analysis

Categorical variables are summarized using frequencies and percentages and compared using a chi-square or Fisher’s exact test as indicated. Continuous variables are described using mean and standard deviation (SD), or median and interquartile range (IQR), as appropriate, and compared using a *t*-test or rank-sum test. Rates are presented as point estimates with associated 95% confidence intervals using the exact Poisson method. Time-to-event outcomes are summarized and visualized using Kaplan–Meier estimates.

Differences in time-to-death between cases and controls were analyzed using a univariable and multivariable marginal Cox model. Adjustments were made for potential confounding variables that could influence the relationship between chronic hypoparathyroidism and mortality. We adjusted for the following factors: age, hypertension, dyslipidemia, type 1 and type 2 diabetes, chronic obstructive pulmonary disease (COPD), ischemic heart disease, atrial fibrillation/flutter, heart failure, valvular heart disease, peripheral vascular disease, and stroke (Supplementary Table S2). Effect sizes were summarized as hazard ratios (HRs) with associated 95% confidence intervals (CI). Hazard proportionality was assessed via analysis of scaled Schoenfeld residuals.

Sensitivity analyses were performed including all 1,982 patients and 19,820 controls, regardless of thyroid cancer status, using the same marginal Cox models as in the primary analysis.

Interaction analyses were performed to evaluate potential effect modification by sex, by adding a sex and hypoparathyroidism interaction term to the Cox models.

For all analyses, *P* < 0.05 was considered significant. All analyses were conducted using Stata version 18 (StataCorp. 2023. *Stata Statistical Software: Release 18*. USA: StataCorp LLC).

## Results

We identified 1,982 patients with chronic hypoparathyroidism during 1997 and 2018. Of these, 71.8% had postsurgical hypoparathyroidism, while 28.2% had nonsurgical hypoparathyroidism. This corresponds to an estimated prevalence of 19.4 per 100,000 inhabitants in Sweden, based on the total population of 10,230,185 inhabitants at the end of inclusion, December 2018 (18). The estimated prevalence of postsurgical hypoparathyroidism was 13.9 per 100,000 (95% CI: 13.2–14.7), while the prevalence of nonsurgical hypoparathyroidism was 5.5 per 100,000 (95% CI: 5.0–5.9).

**Figure 2:**
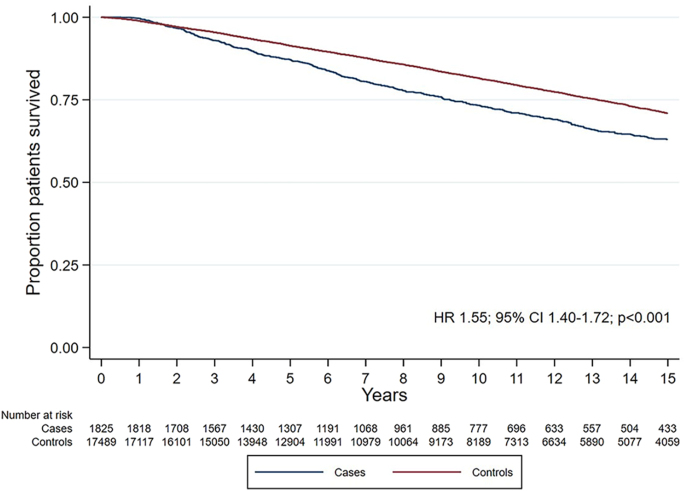
Kaplan–Meier survival curve for adjusted all-cause mortality in patients with hypoparathyroidism compared to matched controls over a 15-year follow-up period.

For all subsequent analyses of mortality, we excluded individuals with baseline thyroid cancer, leaving a total of 1,825 patients and 17,922 matched controls. The mean age (SD) was 51.2 (19.7) years for patients and 50.5 (18.7) years for controls (*P* < 0.001). Most patients (77.5%) were female, and 71.8% had postsurgical chronic hypoparathyroidism (Table 1). The mean follow-up time (SD) was 9.0 (5.0) years for patients and 9.1 (5.1) years for controls (*P* = 0.83). In total, 12 cases and 10 controls received PTH analogs during follow-up. Over the study period, 490 (27%) deaths were recorded among patients and 3,517 (20%) among controls. Patients with hypoparathyroidism had significantly higher all-cause mortality than controls, with an unadjusted hazard ratio of 1.41 (95% CI: 1.31–1.51). Mostly small, but significantly higher baseline prevalence of comorbidities was observed in patients with hypoparathyroidism compared to controls, including hypertension, type 1 and type 2 diabetes, COPD, ischemic heart disease, atrial fibrillation/flutter, heart failure, valvular heart disease, and peripheral vascular disease. No significant differences were found in the baseline prevalence of dyslipidemia or stroke between the two groups (Table 1). Following adjustment for age and comorbidities at baseline, the hazard ratio for all-cause mortality in patients with hypoparathyroidism was 1.55 (95% CI: 1.40–1.72) (Fig. 2). In sensitivity analyses including the full cohort prior to any exclusions, regardless of thyroid cancer status, the association remained statistically significant, although slightly attenuated (adjusted HR: 1.17, 95% CI: 1.07–1.29; Supplementary Table S5).

**Table 1:** Baseline characteristics of patients with chronic hypoparathyroidism and matched controls, after exclusion of thyroid cancer at baseline.

	Cases	Controls	*P*-value*
Number included	1,825	17,922	
Age, years – mean (SD)	51.2 (19.7)	50.5 (18.7)	<0.001
Sex, *n* (%)			0.962
Women	1,414 (77.5)	13,877 (77.4)	
Men	411 (22.5)	4,045 (22.6)	
Follow-up time, years – mean (SD)	9.0 (5.0)	9.1 (5.1)	0.829
Etiology, *n* (%)			
Postsurgical hypoparathyroidism	1,284 (70.4)	N/A	
DiGeorge syndrome	27 (1.4)	N/A	
Autoimmune polyglandular failure	22 (1.1)	N/A	
Idiopathic hypoparathyroidism	227 (12.4)	N/A	
Other or unspecified hypoparathyroidism	265 (14.5)	N/A	
Dispensation of calcium supplementation^†^, *n* (%)	1,479 (74.6)		
Dispensations of calcium supplementation per year, median (IQR)	3.4 (1.2–6.3)		
Dispensations of active vitamin D per year^‡^, median (IQR)	6.2 (4.3–10.2)		
Comorbidities, baseline^§^			
Hypertension, *n* (%)	266 (14.6)	1,641 (9.2)	<0.001
Dyslipidemia, *n* (%)	52 (2.9)	508 (2.8)	0.971
Diabetes type 1, *n* (%)	49 (2.7)	258 (1.4)	<0.001
Diabetes type 2, *n* (%)	79 (4.3)	579 (3.2)	0.013
Chronic obstructive pulmonary disease, *n* (%)	51 (2.8)	245 (1.4)	<0.001
Ischemic heart disease, *n* (%)	112 (6.1)	839 (4.7)	0.006
Atrial fibrillation/flutter, *n* (%)	108 (5.9)	542 (3.0)	<0.001
Heart failure, *n* (%)	64 (3.5)	361 (2.0)	<0.001
Valvular heart disease, *n* (%)	35 (1.9)	183 (1.0)	<0.001
Peripheral vascular disease, *n* (%)	36 (2.0)	194 (1.1)	0.001
Stroke, *n* (%)	59 (3.2)	453 (2.5)	0.071

* *t*-test, chi-square test, or Fisher’s exact test as appropriate.

^†^ Dispensation of calcium at least once per year during the follow-up.

^‡^ Dispensation of active vitamin D per year (ATC codes: A11CC02, A11CC03, A11CC04).

^§^ Any baseline record of an inpatient admission or outpatient episode with listed codes.

Both patients with nonsurgical hypoparathyroidism (HR: 2.16, 95% CI: 1.76–2.65) and those with postsurgical hypoparathyroidism (HR: 1.39, 95% CI: 1.23–1.58) had significantly higher mortality rates compared to matched controls (Supplementary Table S6). No differences in mortality rates were observed between males and females with chronic hypoparathyroidism (*P* for interaction = 0.384).

The leading causes of death among patients with chronic hypoparathyroidism were endocrine, nutritional, and metabolic disorders (HR: 6.46, 95% CI: 2.43–17.21); infectious diseases (HR: 3.53, 95% CI: 2.07–6.04); and diseases of the genitourinary system (HR: 2.40, 95% CI: 1.33–4.33), the respiratory system (HR: 1.67, 95% CI: 1.23–2.28), and the circulatory system (HR: 1.47, 95% CI: 1.23–1.77) (Table 2). Among these, the most common causes of death were infectious diseases, renal failure, and heart failure.

**Table 2:** Causes of death among patients with chronic hypoparathyroidism and matched controls.

Cause of death – *n* (%)	Cases (*n* = 1,825)	Controls (*n* = 17,922)	Unadj HR (95% CI), *P*-value*	Adj HR^†^ (95% CI), *P*-value*
Endocrine, nutritional, metabolic diseases, and metabolic disorders	8 (0.4)	15 (0.1)	5.36 (2.27–12.64), <0.001	6.46 (2.43–17.21), <0.001
Infectious and parasitic diseases	25 (1.4)	86 (0.5)	2.91 (1.86–4.54), <0.001	3.53 (2.07–6.04), <0.001
Diseases of the genitourinary system	22 (1.2)	73 (0.4)	3.02 (1.87–4.86), <0.001	2.40 (1.33–4.33), 0.004
Symptoms, signs, and ill-defined conditions	98 (5.4)	659 (3.7)	1.50 (1.21–1.85), <0.001	1.79 (1.43–2.24), <0.001
Diseases of the respiratory system	57 (3.1)	381 (2.1)	1.51 (1.14–2.00), 0.004	1.67 (1.23–2.28), 0.001
Disease of the circulatory system	173 (9.5)	1,282 (7.2)	1.36 (1.17–1.60), <0.001	1.47 (1.23–1.77), <0.001
Diseases of the digestive system	10 (0.6)	83 (0.5)	1.20 (0.62–2.32), 0.582	1.41 (0.69–2.89), 0.343
Diseases of the nervous system and sense organs	12 (0.7)	115 (0.6)	1.06 (0.59–1.93), 0.843	1.31 (0.65–2.63), 0.444
Neoplasms	65 (3.6)	606 (3.4)	1.09 (0.84–1.41), 0.513	1.15 (0.88–1.51) 0.318
Mental disorders	11 (0.6)	142 (0.8)	0.78 (0.42–1.45), 0.434	0.97 (0.50–1.90), 0.936

* Univariate analysis; reference = controls.

^†^ Adjusted for age and the following baseline comorbidities: hypertension, dyslipidemia, type 1 diabetes, type 2 diabetes, ischemic heart disease, stroke, chronic obstructive pulmonary disease, atrial fibrillation, heart failure, valvular heart disease, and peripheral vascular disease.

**Figure 3:**
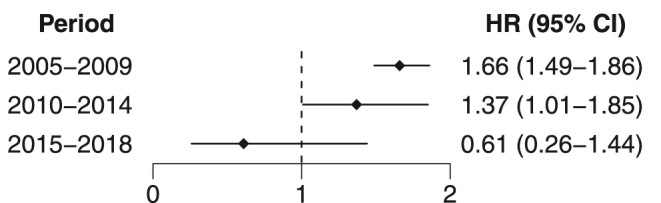
Temporal trends in mortality risk by calendar period of follow-up.

The mortality rate was significantly higher among patients with hypoparathyroidism compared with controls during the periods 2005–2009 (HR: 1.66, 95% CI: 1.49–1.86) and 2010–2014 (HR: 1.37, 95% CI: 1.01–1.85), whereas no significant difference was observed in 2015–2018 (HR: 0.61, 95% CI: 0.26–1.44) (Fig. 3). The distribution of cause-specific mortality among patients and controls across calendar periods is presented in Supplementary Table S4.

## Discussion

In this register-based study, patients with chronic hypoparathyroidism exhibited a significantly higher risk of all-cause mortality compared with matched controls. In sensitivity analyses that included all individuals, regardless of thyroid cancer status, the association remained statistically significant. Patients with nonsurgical hypoparathyroidism had a higher mortality rate than those with postsurgical disease, whereas no sex-related differences in mortality were observed among individuals with chronic hypoparathyroidism.

Our study builds on prior work, including a 2018 study from Sweden by Almquist *et al.*, which reported increased mortality in hypoparathyroidism patients following total thyroidectomy for benign disease (8). Our study utilized refined methodological approaches and provided novel insights (8). We adopted stricter inclusion criteria aligned with current guidelines, requiring active vitamin D treatment for at least 12 months in postsurgical hypoparathyroidism (8, 19). To further ensure the accurate inclusion of only chronic hypoparathyroidism patients, we also required treatment during the last year of follow-up. This approach excluded transient hypoparathyroidism and provided a more accurate analysis of chronic hypoparathyroidism (8). In addition, we included patients with chronic hypoparathyroidism from all etiologies, allowing for a more comprehensive analysis.

Consistent with our findings, a study from Scotland using regional registers reported increased mortality in patients with nonsurgical hypoparathyroidism compared to matched population controls (9). However, that study did not observe increased mortality among patients with postsurgical hypoparathyroidism (9). The discrepancy may be due to differences in disease duration, as nonsurgical hypoparathyroidism often reflects longer-standing disease and is frequently associated with autoimmune or genetic conditions, which carry additional health risks.

In contrast, a recent nationwide study from Denmark focusing on postsurgical hypoparathyroidism reported no association between hypoparathyroidism and mortality following total thyroidectomy for benign disease (11). Similarly, studies from South Korea, focusing on nonsurgical hypoparathyroidism, and a systematic review and meta-analysis on postsurgical hypoparathyroidism, reported no significant mortality differences between hypoparathyroidism patients and controls (12, 20). The systematic review and meta-analysis, which exclusively examined postsurgical cases, found no significant association with mortality. These findings may, however, be limited by smaller sample sizes, exclusive focus on postsurgical cases, and short follow-up durations, with only three of six included studies exceeding five years (20).

Infections, kidney failure, and heart failure were the leading causes of death in this study. The observed elevated risk of mortality due to infections is consistent with prior research suggesting hypoparathyroidism patients may be more susceptible to infections, potentially due to impaired immune function associated with disrupted calcium-phosphate metabolism (9, 19, 21, 22). The strong association between chronic hypoparathyroidism and kidney complications is well established, as recent findings from our group show that patients with chronic hypoparathyroidism have over three times the risk of urolithiasis and more than four times the risk of chronic kidney disease compared to controls (23).

The observed variation in overall mortality rates over time may reflect advancements in clinical management and follow-up, increased awareness of hypoparathyroidism, and more systematic identification and treatment of affected individuals. In addition, survival bias may have contributed, as patients with more severe disease may have died earlier in the observation period. The reduced number of deaths in the latest calendar period may also have resulted in limited statistical power, reducing the ability to detect a significant association.

We estimated the prevalence of chronic hypoparathyroidism in Sweden at 19.4 per 100,000 inhabitants. The reported prevalence aligns closely with data from Denmark, Scotland, and Italy (10, 21, 24, 25) but is nearly double that of Norway (26). The observed proportion of nonsurgical hypoparathyroidism (28%) may appear high compared to the Danish study by Underbjerg *et al.*, but it is almost identical to that reported in Norway and the USA (21, 26, 27). Differences in data sources, such as national health registries vs insurance claim databases, surgical expertise, and variability in the definitions of postsurgical hypoparathyroidism, may account for these discrepancies. Notably, a systematic review of 89 studies, published between 2010 and 2017, identified 20 different definitions of postsurgical hypoparathyroidism (28).

The strengths of this study include its large sample size, facilitated by using nationwide registers, which enabled comprehensive and unbiased data collection of hypoparathyroidism of all etiologies, despite the rarity of hypoparathyroidism. The validity of hypoparathyroidism diagnoses recorded in the Swedish National Patient Register has been confirmed, with a positive predictive value of 91% (29). Moreover, our strict definition of chronic hypoparathyroidism, requiring prolonged active vitamin D treatment, minimized the inclusion of transient cases, enhancing the reliability of our findings. The proportion of nonsurgical hypoparathyroidism in this cohort (28%) is similar to what has previously been reported (26, 27). This study has several limitations. A total of 322 patients were excluded for not receiving at least two dispensations during the final year of follow-up, possibly excluding those treated with low-dose active vitamin D or calcium alone. In Sweden, calcium supplementation is typically prescribed to qualify for reimbursement through the public healthcare subsidy system. As a result, calcium use among patients with chronic hypoparathyroidism is primarily prescription-based, and over-the-counter (OTC) use is likely minimal. However, we acknowledge that some unrecorded OTC use may have occurred and cannot be entirely excluded. Patients who died before the Swedish Prescribed Drug Register started in 2005 were also excluded. Due to these strict inclusion and exclusion criteria, the reported prevalence likely represents a minimal estimate of chronic hypoparathyroidism in Sweden.

Furthermore, some cases of thyroid cancer may have been registered after the index date of hypoparathyroidism or misclassified in the registry, potentially leading to an underestimation of thyroid cancer as an underlying cause of postsurgical hypoparathyroidism.

Identifying cases based solely on ICD codes and prescription data, without access to biochemical markers such as calcium or PTH, is a limitation. This may result in both over- and under-inclusion. To reduce the risk of misclassification, we applied strict and conservative inclusion criteria, requiring consistent diagnostic coding and repeated dispensations of active vitamin D. While this likely excluded patients with milder disease or those not receiving conventional treatment, it also minimized the risk of including individuals without true chronic hypoparathyroidism.

Although patients and controls were matched according to the first recorded date of diagnosis, some patients, especially those with nonsurgical causes, may have had hypoparathyroidism for several years before it was diagnosed or officially documented. There is also a potential risk that some of the patients with the diagnosis code ‘other and unspecified’ hypoparathyroidism were misclassified. The lack of access to medical records and biochemical verification remains a limitation and prevents assessment of optimal biochemical control and its potential impact on outcomes. Another consideration is the possible inclusion of patients who developed chronic kidney disease or malabsorption during follow-up and received active vitamin D for reasons unrelated to hypoparathyroidism. To reduce this risk, we excluded individuals with known chronic kidney disease at baseline, but misclassification remains possible over the long observation period. In addition, the Swedish National Patient Register is limited to inpatient and outpatient hospital-based care, potentially excluding patients and controls managed exclusively in primary care. This could result in an underestimation of comorbidities or the exclusion of individuals whose care did not involve hospital settings. However, in Sweden, patients with hypoparathyroidism are followed up by endocrinologists in specialist care, according to our clinical guidelines, and are therefore likely to be captured in the register. Furthermore, although our analyses were adjusted for a wide range of relevant comorbidities, the association between chronic hypoparathyroidism and increased mortality remained significant. This suggests that the observed association is not only due to comorbidity burden. However, the possibility of residual confounding due to unmeasured or misclassified comorbidities cannot be fully excluded.

Although controls were matched by year of birth, a small age difference remained, likely due to calendar-year matching and exclusions made after matching. This was adjusted for in the analyses and is unlikely to have impacted the results, but we acknowledge this as a potential limitation.

PTH analogs have not been part of standard care for chronic hypoparathyroidism in Sweden. Only a very small number of individuals (*n* = 22; 12 cases and 10 controls, 0.1%) received such treatment during the study period and were therefore unlikely to have affected the modeling results. Finally, while the Swedish Cause of Death Register is a high-quality data source, distinguishing between immediate and underlying causes of death remains inherently subjective, which may introduce classification bias (16).

Our findings demonstrate that patients with chronic hypoparathyroidism receiving conventional treatment in Sweden have a higher mortality rate compared to matched controls. This underscores the substantial clinical burden of the disease, particularly in nonsurgical cases. Furthermore, it highlights the importance of clinician awareness of its long-term complications and supports the need for continued evaluation of disease management in this population. Further research is needed to explore whether optimized therapy and biochemical control can improve long-term outcomes.

## Supplementary materials













## Declaration of interest

WK received lecture fees from UCB Pharma AB. MM has institutional research grants from and is an advisor to Takeda, BridgeBio, and Amolyt. TS served on scientific advisory boards for Biogen and received consultancy fees from AbbVie. OK owns shares in Navinci Diagnostics AB, Uppsala, Sweden. SB received an institutional research grant from Shire. The remaining authors declare no conflicts of interest.

## Funding

This study was funded by research grants from the Swedish Research Council, the Knut and Alice Wallenberg Foundation, the Novo Nordisk Foundation, the Torsten and Ragnar Söderberg Foundation, and the Kristian Gerhard Jebsen Foundation.

## Ethical approval

Ethical approval for this nationwide registry-based study was obtained from the Swedish Ethical Review Authority in Stockholm, Sweden (approval number: 2017/476–31/4).
