# Supplementary material for: Increased mortality in chronic hypoparathyroidism: a nationwide cohort study in Sweden
Source: Endocr Connect. 2026 Jul 7;15(7):e250450. doi: 10.1530/EC-25-0450 (PMC13383239; doi:10.1530/EC-25-0450)
Supplement: Supplementary file 1 [file EC-25-0450_supplementary_table_s1.pdf]

Supplementary Table S1. ICD-codes used for identifying different etiologies of hypoparathyroidism

	ICD-10 diagnoses
Postsurgical hypoparathyroidism	E89.2
DiGeorge syndrome	D82.1
Autoimmune polyglandular failure	E31.0
Idiopathic hypoparathyroidism	E20.0
Other or unspecified hypoparathyroidism	E20.2-E20.9
