# Supplementary material for: Increased mortality in chronic hypoparathyroidism: a nationwide cohort study in Sweden
Source: Endocr Connect. 2026 Jul 7;15(7):e250450. doi: 10.1530/EC-25-0450 (PMC13383239; doi:10.1530/EC-25-0450)
Supplement: Supplementary file 2 [file EC-25-0450_supplementary_table_s2.pdf]

Supplementary Table S2. ICD-codes used for adjustment of comorbidities

	ICD-10 diagnoses
Hypertension	I10-I15
Dyslipidemia	E78
Diabetes Type 1	E10
Diabetes Type 2	E11
Chronic Obstructive Pulmonary Disease	J44
Ischemic Heart Disease	I20-I25
Atrial Fibrillation/Flutter	I48
Heart Failure	I50
Valvular Heart Disease	I05-I08, I34-I39
Peripheral Vascular Disease	I70-I73
Stroke	I60-I64, I69
