# Supplementary material for: Increased mortality in chronic hypoparathyroidism: a nationwide cohort study in Sweden
Source: Endocr Connect. 2026 Jul 7;15(7):e250450. doi: 10.1530/EC-25-0450 (PMC13383239; doi:10.1530/EC-25-0450)
Supplement: Supplementary file 3 [file EC-25-0450_supplementary_table_s3.pdf]

Supplementary Table S3. Baseline comorbidities of patients with postsurgical and nonsurgical hypoparathyroidism (hypoPT)

	All patients (n=1,825)	Postsurgical hypoPT (n=1,284)	Nonsurgical hypoPT (n=541)	P-value*
**Comorbidities, baseline[1]**				
Hypertension, n (%)	266 (14.6)	181 (14.1)	85 (15.7)	0.372
Dyslipidemia, n (%)	52 (2.9)	31 (2.4)	21 (3.9)	0.085
Diabetes Type 1, n (%)	49 (2.7)	25 (2.0)	24 (4.4)	0.003
Diabetes Type 2, n (%)	79 (4.3)	50 (3.9)	29 (5.4)	0.160
Chronic Obstructive Pulmonary Disease, n (%)	51 (2.8)	36 (2.8)	15 (2.8)	0.971
Ischemic Heart Disease, n (%)	112 (6.1)	65 (5.1)	47 (8.7)	0.003
Atrial Fibrillation/Flutter, n (%)	108 (5.9)	73 (5.7)	35 (6.5)	0.517
Heart Failure, n (%)	64 (3.5)	39 (3.0)	25 (4.6)	0.093
Valvular Heart Disease, n (%)	35 (1.9)	24 (1.9)	11 (2.0)	0.815
Peripheral Vascular Disease, n (%)	36 (2.0)	23 (1.8)	13 (2.4)	0.391
Stroke, n (%)	59 (3.2)	38 (3.0)	21 (3.9)	0.309

1. *Any baseline record of an inpatient admission or outpatient episode with listed codes*

**Chi-square test*
