# Supplementary material for: Increased mortality in chronic hypoparathyroidism: a nationwide cohort study in Sweden
Source: Endocr Connect. 2026 Jul 7;15(7):e250450. doi: 10.1530/EC-25-0450 (PMC13383239; doi:10.1530/EC-25-0450)
Supplement: Supplementary file 4 [file EC-25-0450_supplementary_table_s4.pdf]

Supplementary Table S4. Causes of death among patients with chronic hypoparathyroidism and matched controls.

	2005-2009		2010-2014		2015-2018	
**Cause of death - n (%)**	Cases (n=1,062)	Controls (n=10,301)	Cases (n=428)	Controls (n=4,274)	Cases (n=335)	Controls (n=3,347)
**Endocrine, nutritional, metabolic diseases and metabolic disorders**	7 (0.7)	12 (0.1)	1 (0.2)	2 (0.1)	0 (0.0)	1 (0.0)
**Infectious and parasitic diseases**	20 (1.9)	62 (0.6)	5 (1.2)	18 (0.4)	0 (0.0)	6 (0.2)
**Diseases of the genitourinary system**	22 (2.1)	65 (0.6)	0 (0.0)	8 (0.2)	0 (0.0)	0 (0.0)
**Symptoms, signs, and ill-defined conditions**	81 (7.6)	554 (5.4)	14 (3.3)	82 (1.9)	3 (0.9)	23 (0.7)
**Diseases of the respiratory system**	49 (4.6)	311 (3.0)	8 (1.9)	67 (1.6)	0 (0.0)	3 (0.1)
**Disease of the circulatory system**	148 (13.9)	1039 (10.1)	24 (5.6)	198 (4.6)	1 (0.3)	45 (1.3)
**Diseases of the digestive system**	7 (0.7)	69 (0.7)	3 (0.7)	11 (0.3)	0 (0.0)	3 (0.1)
**Diseases of the nervous system and sense organs**	9 (0.9)	89 (0.9)	3 (0.7)	23 (0.5)	0 (0.0)	3 (0.1)
**Neoplasms**	48 (4.5)	500 (4.9)	12 (2.8)	86 (2.0)	5 (1.5)	20 (0.6)
**Mental disorders**	8 (0.8)	117 (1.1)	2 (0.5)	18 (0.4)	1 (0.3)	7 (0.2)
