# Supplementary material for: Increased mortality in chronic hypoparathyroidism: a nationwide cohort study in Sweden
Source: Endocr Connect. 2026 Jul 7;15(7):e250450. doi: 10.1530/EC-25-0450 (PMC13383239; doi:10.1530/EC-25-0450)
Supplement: Supplementary file 5 [file EC-25-0450_supplementary_table_s5.pdf]

Supplementary Table S5. All-cause mortality in patients with chronic hypoparathyroidism compared with matched controls: primary analysis excluding thyroid cancer and sensitivity analysis including all individuals

	**Primary analysis:** Cases (n=1,825) Controls (n=17,922)		**Sensitivity analysis:** Cases (n=1,982) Controls (n=19,820)	
	Unadjusted HR (95% CI)	Adjusted HR[1] (95% CI)	Unadjusted HR (95% CI)	Adjusted HR[1] (95% CI)
Hypoparathyroidism	1.41 (1.31-1.51)	1.55 (1.40-1.72)	1.33 (1.21-1.46)	1.17 (1.07-1.29)

[1] Adjusted for baseline comorbidities: hypertension, dyslipidaemia, type 1 diabetes, type 2 diabetes, ischemic heart disease, stroke, chronic obstructive pulmonary disease, atrial fibrillation, heart failure, valvular heart disease and peripheral vascular disease.
