# Supplementary material for: Increased mortality in chronic hypoparathyroidism: a nationwide cohort study in Sweden
Source: Endocr Connect. 2026 Jul 7;15(7):e250450. doi: 10.1530/EC-25-0450 (PMC13383239; doi:10.1530/EC-25-0450)
Supplement: Supplementary file 6 [file EC-25-0450_supplementary_table_s6.pdf]

Supplementary Table S6. All-cause mortality in patients with chronic hypoparathyroidism subgroup analysis by etiology, compared to matched controls.

Subgroup	Adjusted HR (95% CI)
Postsurgical hypoparathyroidism[1]	1.39 (1.23-1.58)
Nonsurgical hypoparathyroidism[1]	2.16 (1.76-2.65)

[1] Adjusted for age and baseline comorbidities: hypertension, dyslipidaemia, type 1 diabetes, type 2 diabetes, ischemic heart disease, stroke, chron obstructive pulmonary disease, atrial fibrillation, heart failure, valvular heart disease and peripheral vascular disease.
